# The impact of COVID-19 on surgical practice in Jordan during the second outbreak: A survey^☆^

**DOI:** 10.1016/j.amsu.2021.01.047

**Published:** 2021-01-29

**Authors:** Raed M. Ennab, Rasheed K. Ibdah

**Affiliations:** a Department of Clinical Sciences/Vascular Surgery, Faculty of Medicine, Yarmouk University, Irbid, Jordan; b Department of Internal Medicine, Faculty of Medicine, Jordan University of Science and Technology, Irbid, Jordan

**Keywords:** COVID-19, SARS-CoV-2, Survey, Operations, Hospital, Jordan

## Abstract

**Background:**

COVID-19 is an acute respiratory pandemic with no available effective antiviral treatment or widely available effective vaccine. Surgical practice has faced widespread problems due to the pandemic including viral transmission risk and cross-infection, staffing problems, prioritizations of surgical procedures, and lack of beds due to occupancy of hospitals and ICU beds with COVID-19 patients.

**Methods:**

A survey was conducted between October 31 to November 4, 2020, through google forms. The questionnaire involved 16 questions sent to consultants and specialists of all general and special surgical specialties and subspecialties in Jordan.

**Results:**

We have got responses from surgeons of all public and private sectors in Jordan. There was a pronounced decline in the number of elective and emergency procedures performed during October 2020 due to COVID-19 pandemic related reasons.

**Conclusions:**

The impact of COVID-19 on the surgical practice in Jordan during October 2020 was moderate to prominent. Measures that could be made to alleviate this impact include the assignment of certain hospitals for covid-19 patients as a step before the establishment of field hospitals and the cooperation between the private and the public health sectors.

## Highlights

• Surgical practice in Jordan faced widespread problems due to the COVID-19 pandemic.
• The total number of confirmed cases in Jordan raised from 2097 to 120982 cases between September and November 2020.
• All health sectors in Jordan were affected by the pandemic.
• There was a pronounced decline in the number of elective and emergency cases performed during October 2020 in Jordan.

## 1 Introduction

COVID-19 is mainly an acute respiratory syndrome caused by the severe scute Respiratory syndrome Coronavirus-type 2 (SARS-CoV-2) which is a highly contagious virus [1]. The world health organization (WHO) declared it as a pandemic in March 2020 after it had spread in the world and lead to significant effects on all aspects of human life [2]. Up to November 2020, there is no effective antiviral drug treatment for COVID-19, and no widely available effective vaccine [3,4].

**Fig. 1:**
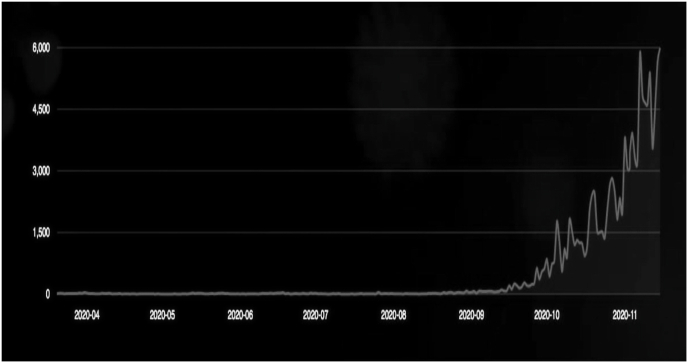
A chart of the new cases of COVID-19 reported each day in Jordan by the Jordanian Ministry of health between March 15 and November 19, 2020 [8].

Jordan is a middle eastern country with a population of 10.77 million [5]. The government has followed the recommendations released by the WHO and led the battle against the virus through a multi-disciplinary team at the National Center for Security and Crises Management (NCSCM) [6]. An early measure to control the infection was a lockdown for one and a half months starting on the March 15, 2020 [7]. This had led to a great success in the control of the disease for a few months thereafter with a low number of cases (Fig. 1) [8]. Unfortunately, the lockdown had a negative impact on the economy so new measures were taken with the resumption of sectors and reopening to the world under strict control and preventive measures. However, despite all the health measures and governmental and security efforts the disease spread across the country in parallel with the second outbreak in the world which started in September. The total number of confirmed cases raised from 2097 to 120,982 cases between the 1st of September to the November 10, 2020, and the daily number of confirmed reported cases raised from few cases to about 5000 cases each day (Fig. 1) with total deaths of 1386 patients due to COVID-19 since the beginning of the pandemic [8,9].

The Surgical practice has faced widespread problems due to the pandemic including viral transmission risk and cross-infection, staffing problems, prioritizations of surgical procedures, and lack of beds due to occupancy of hospitals and ICU beds with COVID-19 patients [10]. In this study, we conducted a survey to consultants and specialists of different surgical specialties in Jordan to estimate the impact of the second outbreak on the surgical operations as an objective measure and indicator of the impact on health services. This work has been reported in line with the STROCSS criteria for cross-sectional studies [11].

## 2 Materials and methods

A survey was conducted between October 31 and November 4, 2020. The questionnaire was made through google forms, and a consent was taken from the Jordan medical association to do the research. The survey link was sent to surgeons in Jordan through social media including Facebook related groups and pages and Whatsapp private and group messages. This research was registered at ClinicalTrials.gov with the registration ID (NCT04650035) in accordance with the Declaration of Helsinki 2013 of research involving human participants [12]. The survey was introduced with the invitation to participate of surgeons of all general and special surgical specialties and subspecialties. The aim of the survey was mentioned in the introduction which was to evaluate the impact of COVID-19 on surgical practice in Jordan during the second outbreak of COVID-19, and it was explained that the aim of the survey was for research purposes only and that it was anonymous with privacy preserved, and no identifying information required. Also, it was noted that we have added spaces for written views and recommendations.

The questionnaire involved 16 questions of the multiple-choice, the dropdown, the multiple check, and the short answer questions (Table 1). The questions were decided upon by the authors to achieve the aim of the study, and compare the number of surgeries done during October 2020 to the average monthly rate during 2019, and check the reasons for any differences and the recommendations of the surgeons based upon their experience, which may benefit the health authorities. The first 2 questions were about the specialty and the place of work whether it was the ministry of health, the royal medical services, the university hospitals, or the non-governmental organizations. The 3rd and 4th questions were about the hospital where the participant works whether it admits COVID-19 patients and/or ordinary patients.

**Table 1:** The questions of the survey and the options for the answers.

Q1. Where do you work? *
• Choose (out of a dropbox)
Q2. What is your surgical specialty? *
•Choose (out of a dropbox)
Q3. Does your hospital admit COVID-19 patients? *
•Yes
•No
Q4. Does your hospital admit ordinary (non-covid19) patients? *
•Yes
•No
Q5. What was the average monthly rate of elective surgeries you did in 2019? *
•Your answer
Q6. What was the average monthly rate of emergency surgeries you did in 2019? *
•Your answer
Q7. How many elective surgeries you did in October 2020? *
•Your answer
Q8. How many emergency surgeries you did in October 2020? *
•Your answer
Q9. Did you cancel any elective procedure for coronavirus reasons in October 2020? *
•Yes
•No
Q10. If yes, please specify the reasons:
•Less beds are available due to occupancy by COVID-19 patients.
•Patients were postponed because they were required to do a coronavirus test.
•Some patients were found to have a positive test
•Patients chose to postpone their surgeries for fear of getting infected in the hospital.
•Less staff at the hospital because some of them had positive tests
•the department was locked down temporarily for disinfection
•You have chosen to reduce or cancel elective procedures for fear of cross-infection.
•Other:
Q11. If yes, how many elective procedures have you canceled?
•Your answer
Q12. Did you have a problem admitting any emergency patient for coronavirus reasons in October 2020? *
•Yes
•No
Q13. If yes, please specify the reasons:
•No available beds due to occupancy by COVID-19 patients.
•the patient tested positive for coronavirus
•Other:
Q14. If yes, how many times did you have a problem admitting any emergency patient?
•Your answer
Q15. The effect of COVID-19 on your practice during the last month was: *
•Prominent
•Moderate
•Mild
•No effect
Q16. What are your recommendations?
•To establish special hospitals for COVID-19.
•To assign certain hospitals for COVID-19
•To assign certain departments in hospitals for COVID-19
•To postpone all elective patients for fear of cross-infection
•Other:

The Questions from the 5th to the 8th were about the volume of work in terms of the number of elective and emergency surgeries during October 2020 in comparison to the average monthly rate during the year 2019. The questions from the 9th to the 15th were about the problems that might have been faced in managing elective and emergency cases during October 2020 in relation to COVID-19. The last question was a multiple checkbox question with an added space for giving the possible recommendations to manage the situation.

## 3 Results

A total of 60 responses were received from specialists and consultants of different surgical specialties and subspecialties. There were 26 (43.3%) surgeons from the ministry of health, 13 (21.7%) surgeons from the private sector, 12 (20%) surgeons from the royal medical services, and nine (15%) surgeons from university hospitals (Table 2). Most responses were general surgeons (19; 31.7%) followed by orthopedic surgeons (13; 21.7%). Other specialties contributions are shown in Table 2.

**Table 2:** The answers to the first and second questions about the specialty and the place of work and the number and the percentage of responses for each answer.

Q1. Where do you work?	N. (pct.)
The ministry of health	26 (43.3%)
Royal medical services	12 (20%)
University hospitals	9 (15%)
Private hospital/Clinic	13 (21.7%)
Non-governmental organizations	0
**Q2. What is your surgical specialty?**	**N. (pct.)**
General Surgery	19 (31.7%)
Upper GI	1 (1.7%)
Colorectal	0
Vascular	4 (6.7%)
Cardiac	5 (8.3%)
Thoracic	1 (1.7%)
Plastic	1 (1.7%)
Head and Neck	0
Endocrine	0
Oncosurgery	0
Urology	3 (5%)
Orthopedic	13 (21.7%)
Neurosurgery	0
ENT	6 (10%)
Maxillofacial	0
Obstetrics and Gynecology	4 (6.7%)
Ophthalmology	3 (5%)

The number of surgeons who answered that their hospitals admitted covid-19 patients was 50 (83.3%), and those who answered that their hospitals admitted ordinary patients were 57 (95%). This means that there were three surgeons who worked at hospitals that admit only COVID-19 patients and no ordinary patients, and that most hospitals involved admitted both covid-19 and non-COVID-19 patients. Questions 5 and 7 were about the monthly rate of elective surgeries during 2019 and the number of elective surgeries performed in October 2020 respectively. Overall there was a dramatic reduction in the number during October 2020 to 48.5% of the usual average monthly rate of 2019. In questions 9 and 10, there were 36 (60%) surgeons who reported that they have canceled elective surgeries for COVID-19 related reasons, also they checked or wrote down the reasons (Table 3).

**Table 3:** The answers to question number 10 about the reasons for canceling elective procedures in relation to COVID-19.

Reasons for canceling elective surgeries	N. (pct.)
• Less beds are available due to occupancy by COVID-19 patients.	16 (44.4%)
•Patients were postponed because they were required to do a coronavirus test.	8 (22.2%)
•Some patients were found to have a positive test	12 (33.3%)
•Patients chose to postpone their surgeries for fear of getting infected in the hospital.	15 (41.7%)
•Less staff at the hospital because some of them had positive tests.	11 (30.6%)
•the department was locked down temporarily for disinfection.	9 (25%)
•You have chosen to reduce or cancel elective procedures for fear of cross-infection.	19 (52.8%)
•Lack of ICU beds sometimes needed for certain patients post-operatively.	1 (2.8%)
•The doctor had a coronavirus.	1 (2.8%)
•The anesthetist canceled the surgery even though the patient is well prepared and negative covid-19 due to fear and feeling of disappointment in the pandemic.	1 (2.8%)

Questions 6 and 8 were about the monthly rate of emergency surgeries during 2019 and the number of emergency surgeries performed in October 2020 respectively. Overall there was a dramatic reduction in the number during October 2020 to 64.2% of the usual average monthly rate of 2019. Although 2 orthopedic surgeons from the ministry of health reported an increase by 150% in their emergency surgery operations during October 2020. In questions12, 13, and 14 There were 23 (38.3%) surgeons who answered that they had problems in admitting emergency cases for COVID-19 reasons, and there were 26 surgeons who checked or wrote down the problems (Table 4). The number of patients who faced problems in admission for each surgeon varied between null and 30 emergency cases during October 2020.

**Table 4:** The answers to question number 13 about the problems faced in admitting emergency cases in relation to COVID-19.

Problems in admitting emergency patient	N. (pct.)
•No available beds due to occupancy by COVID-19 patients.	17 (65.4%)
•The patient tested positive for coronavirus	7 (26.9%)
•Waiting for the test result which took more than a day	1 (3.8%)
•Large number of elective patients	1 (3.8%)
•No ICU beds available	1 (3.8%)

Question 15 was about the severity of the effect of COVID-19 on the surgeon's practice. 25 (41.7%) answered that the effect was prominent, and 26 (43.3%) answered that it was moderate. On the other hand, eight (13.3%) responded that the effect was mild and only 1 (1.7%) said that there was no effect.

Question 16 was about the recommendations of the surgeons and there were 58 surgeons who checked or wrote down recommendations (Table 5).

**Table 5:** The answers to question number 16 about the recommendations of the participants.

Recommendations	N. (pct.)
•To establish special hospitals for COVID-19.	42 (72.4%)
•To assign certain hospitals for COVID-19	25 (43.1%)
•To assign certain departments in hospitals for COVID-19	12 (20.7%)
•To postpone all elective patients for fear of cross-infection	20 (34.5%)
•To establish hire staff free for corona period only and fast tests for patients and staff on a regular basis	1 (1.7%)
•To Increase beds and staff numbers and decrease shifts time	1 (1.7%)
•To make tests for all admitted patients	1 (1.7%)
•To establish field hospitals in governorates special for covid-19 patients	1 (1.7%)

## 4 Discussion

The results showed that all health sectors in Jordan including the public and the private sectors were affected by the pandemic. Many hospitals in the private and the public sectors admit COVID-19 patients in isolated departments in addition to admitting ordinary patients. On the other hand, only few hospitals were limited to COVID-19 patients. On October 31, 2020, the total cumulative number of confirmed cases in Jordan was 72,607, and the number of admitted COVID-19 patients was 1542 with 274 patients in the ICUs [8,9]. These large numbers led to a shortage of ICU and floor beds available to manage non-COVID patients. During the first outbreak, the surgical societies of different surgical specialties had released case triage guidelines and procedural prioritization classifications into elective, semi-elective, and emergency [13]. These guidelines recommend cancellation and/or postponing elective cases and giving priority to emergency cases due to shortage of medical staff and facilities and fear of cross-infection. The presence of both COVID and non-COVID patients in the same hospital may increase the chance of transmission of the virus to the staff and the non-infected patients despite all measures and precautions. The infection of the staff leads to a shortage of the medical working force and further negative impact on the health services.

The results of the survey have shown a pronounced decline in the number of elective and emergency cases performed during October in comparison to the average rate of 2019. The governmental sectors started by the end of October 2020 to close outpatient clinics and cancel elective operation lists in a measure to control the transmission of the infection.

There were 23 (38.3%) surgeons who had problems in admitting emergency cases, mostly due to the overwhelming of hospitals and the shortage of beds, and in seven cases the patients were found to be positive for the coronavirus. Emergency surgery on patients who test positive for coronavirus needs special care with the application of special protocols and proper training of the personnel [14]. These protocols are available nowadays in Jordanian hospitals which care for COVID-19 patients.

The largest percentage of surgeons recommended either to establish or to assign special hospitals for COVID-19 patients. This has the advantage of a more secure isolation and separation between ordinary patients and their caring staff from COVID-19 patients, and this may decrease the risk of cross-infection and offers a more secure environment for non-COVID patients.

The limitation of this study is that it was a broad spectrum involving all surgical specialties without differentiation of the type of operation other than being elective or emergency. Further studies for each surgical specialty with more specific details may give results on the impact of delay of surgical procedures due to COVID-19 on morbidity and mortality.

## 5 Conclusions

The impact of COVID-19 on the surgical practice in Jordan during October 2020 was moderate to prominent. Measures that could be made to alleviate this impact include the assignment of certain hospitals for covid-19 patients as a step before the establishment of field hospitals and the cooperation between the private and the public health sectors.

## Provenance and peer review

Not commissioned, externally peer-reviewed.

## Ethical approval

None.

Not needed in this survey because it involved doctors not patients.

## Sources of funding

No sources of funding of the research.

## Author contribution

Study conception and design: Ennab.

Acquisition of data: Ennab, Ibdah.

Analysis and interpretation of data: Ennab, Ibdah.

Drafting of manuscript: Ennab.

Critical revision and Approval: Ennab, Ibdah.

## Conflicts of interest

Authors declare no conflict of interest.

## Registration of research studies

1.Name of the registry: ClinicalTrials.gov.

2.Unique Identifying number or registration ID: NCT04650035.

3.Hyperlink to your specific registration (must be publicly accessible and will be checked): https://clinicaltrials.gov/ct2/show/NCT04650035.

## Guarantor

Raed M Ennab.

Faculty of medicine, department of clinical sciences, Yarmouk University.

Pr. Hasan St. 21,163, Irbid, Jordan.

Tel: +962797122559.

Email: raed.ennab@yu.edu.jo.

## Authors contribution

•Study conception and design: Ennab.

•Acquisition of data: Ennab, Ibdah.

•Analysis and interpretation of data: Ennab, Ibdah.

•Drafting of manuscript: Ennab.

•Critical revision and Approval: Ennab, Ibdah.

## Conflict of interest

The authors declare no conflict of interest.

## Funding

This research received no external funding.
